# Alpha‐Ketoglutarate Ameliorates Synaptic Plasticity Deficits in APP/PS1 Mice Model of Alzheimer's Disease

**DOI:** 10.1111/acel.70235

**Published:** 2025-09-17

**Authors:** Sheeja Navakkode, Brian K. Kennedy

**Affiliations:** ^1^ Healthy Longevity Translational Research Program, Yong Loo Lin School of Medicine, Centre for Healthy Longevity, National University Health System National University of Singapore Singapore Singapore; ^2^ Life Sciences Institute Neurobiology Programme, Centre for Life Sciences National University of Singapore Singapore Singapore; ^3^ Department of Physiology, Yong Loo Lin School of Medicine National University of Singapore Singapore Singapore; ^4^ Department of Biochemistry, Yong Loo Lin School of Medicine National University of Singapore Singapore Singapore; ^5^ Buck Institute for Research on Ageing Novato California USA

**Keywords:** alpha‐ketoglutarate, Alzheimer's disease, autophagy, CaAKG, CP‐AMPA, hippocampus, long‐term potentiation, NMDA, rapamycin, synaptic plasticity

## Abstract

Alzheimer's disease (AD) is one of the most prevalent neurodegenerative disorders, characterized by a progressive decline in cognitive function. Increasing evidence indicates that alpha‐ketoglutarate (AKG), a key metabolite in the tricarboxylic acid (TCA) cycle, can extend lifespan and healthspan across various animal models, raising interest in its potential neuroprotective effects in age‐related disorders such as AD. Our previous research found that dietary supplementation with calcium alpha‐ketoglutarate (CaAKG), a calcium derivative of AKG, enhances both lifespan and healthspan in mice. However, little is known about the neuroprotective role of AKG/CaAKG in AD. Here, we show that CaAKG could rescue synaptic deficits that are associated with AD. Treatment with AKG or CaAKG ameliorates long‐term potentiation (LTP) at hippocampal CA1 synapses in APP/PS1 mice, with a more profound effect in female AD mice than in males. The effects of CaAKG were mediated through an NMDA receptor‐independent mechanism involving L‐type calcium channels (LTCC) and calcium‐permeable AMPA receptors (CP‐AMPARs). Analysis of protein expression showed that AD hippocampal slices treated with CaAKG exhibited increased LC3‐II levels, indicating enhanced autophagy. Similarly, rapamycin, an mTOR inhibitor, also rescued LTP deficits in AD mice, suggesting that the observed increase in autophagy may contribute to neuroprotection. Interestingly, rapamycin showed differential effects, as it rescued LTP in AD mice but blocked LTP in WT mice. We also observed that CaAKG facilitated synaptic tagging and capture (STC), a widely studied cellular model for associative memory, indicating its potential to facilitate associative memory. Overall, our findings suggest that CaAKG has neuroprotective effects in APP/PS1 mice. We propose CaAKG as a promising therapeutic target not only for aging but also for AD and potentially other age‐associated neurodegenerative diseases, highlighting geroprotective strategies as viable alternatives for the prevention and treatment of AD.

## Graphical abstract

This schematic illustration summarizes the effects of calcium alpha‐ketoglutarate (CaAKG) on synaptic plasticity in a mouse model of Alzheimer's disease (APP/PS1). CaAKG enhances autophagy and restores LTP in APP/PS1 mice via NMDAR‐independent, LTCC, and CPAMPAR‐dependent pathways. It also facilitates synaptic tagging and capture, supporting associative memory‐related processes.

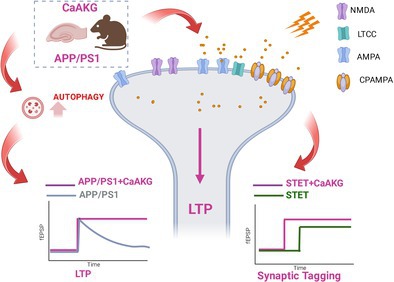

## 1 Introduction

Alpha‐ketoglutarate (AKG), a key metabolite in the tricarboxylic acid (TCA) cycle, is known to extend lifespan in various animal models, including yeast, flies, nematodes, and mice (Asadi Shahmirzadi et al. 2020; Chin et al. 2014; Gyanwali et al. 2022; Naeini et al. 2023). It is a multifunctional molecule, exerting its effects on lifespan by inhibiting ATP synthase and mTOR (Chin et al. 2014), activation of AMP‐activated protein kinase (AMPK) (Su et al. 2019), modulating metabolism, reducing inflammation (Asadi Shahmirzadi et al. 2020), as an antioxidant (Liu et al. 2018), and functioning as an epigenetic modifier (Dhat et al. 2023), among other potential mechanisms.

Moreover, reductions in alpha‐ketoglutarate dehydrogenase (KGDHC) activity are associated with age‐related neurodegenerative diseases like AD and Parkinson's disease (PD) (Gibson et al. 1998; Satpute et al. 2013). KGDHC, the rate‐limiting step in the TCA cycle, and its substrate, AKG, serve as a regulatory centre for multiple signaling processes (Hansen and Gibson 2022). Inactivation of KGDHC by oxidative stress and compounds like 1‐methyl‐4‐phenylpyridinium (MPP^+^), which induce neurodegeneration, further underscores its involvement in AD (Hansen and Gibson 2022; Wiemerslage et al. 2013). Additional evidence supporting its role in AD includes the observation that certain brain regions enriched in choline acetyltransferase, a biomarker for early detection of AD, also have high levels of KGDHC and are sensitive to KGDHC defects (Calingasan et al. 1994; Kim et al. 2006).

Ca‐AKG, a calcium derivative of AKG, has emerged as a promising compound with potential benefits for aging and neurodegenerative diseases (Asadi Shahmirzadi et al. 2020; Zhang et al. 2023). Our previous work demonstrated that Ca‐AKG supplementation extends lifespan and improves healthspan in mice (Asadi Shahmirzadi et al. 2020). Compared to free AKG, Ca‐AKG offers several advantages, including greater chemical stability and improved bioavailability. Ca‐AKG is acidic and absorbed more slowly in the gastrointestinal tract, resulting in a gradual and sustained release into the bloodstream. This prolonged absorption profile enables Ca‐AKG to maintain its biological effects over an extended period, potentially enhancing its overall efficacy. Furthermore, AKG is a charged molecule with limited ability to cross cell membranes (Wu et al. 2016). Given these properties, Ca‐AKG represents a practical and more effective candidate to investigate for its effects on synaptic plasticity and cognitive deficits associated with neurodegenerative diseases (Gyanwali et al. 2022).

The neuropathological hallmarks of AD include extracellular amyloid beta (Aβ) plaques and neurofibrillary tangles (NFTs) consisting of abnormally hyperphosphorylated tau protein (Jellinger 2020; Serrano‐Pozo et al. 2011). However, synaptic dysfunction appears to correlate more strongly with memory loss than these histopathologic hallmarks (Meftah and Gan 2023; Sheng et al. 2012). The synapse is a critical structure, and recovering synaptic dysfunction has been a major target for therapeutic interventions (Jackson et al. 2019). These strategies include boosting long‐term potentiation (LTP), reducing long‐term depression (LTD), maintaining excitatory/inhibitory (E/I) balance, and modulating NMDARs, among others (Blum and Levi 2024; Jackson et al. 2019; Pavon et al. 2024; Siddiqui et al. 2023).

LTP is one of the most widely used cellular models for studying mechanisms of hippocampal synaptic plasticity and learning and memory. It is defined as the persistent increase in synaptic strength following an afferent activity (Bliss and Collingridge 1993; Bliss and Lomo 1973). Altered LTP and synaptic plasticity are observed in several neurodegenerative disorders, including animal models of AD (Ho et al. 2020; Navakkode et al. 2021; Sasaguri et al. 2022). The magnitude and strength of LTP are indicators of cognitive function, making it a target for therapeutics aimed at preserving synaptic plasticity and memory (Jackson et al. 2019; Selkoe 2002).

LTP induction activates NMDARs, allowing calcium (Ca^2+^) to enter into postsynaptic cells, which then activates Ca^2+^‐dependent kinases and promotes AMPAR insertion, enhancing excitatory synaptic transmission (Bliss and Collingridge 1993). Ca^2+^ influx for sustained potentiation can also occur through voltage‐gated Ca^2+^ channels, Ca^2^‐permeable AMPAR (CP‐AMPARs), and intracellular stores like the endoplasmic reticulum and mitochondria (Koek et al. 2024; Li et al. 2017; Malenka and Bear 2004; Patergnani et al. 2011). GluA2‐containing AMPARs are Ca^2+^‐impermeable, whereas GluA2‐lacking CP‐AMPARs allow Ca^2+^ influx (Cull‐Candy and Farrant 2021) (Isaac et al. 2007). CP‐AMPARs amplify synaptic signals, so even minor changes in their expression can significantly affect synaptic transmission (Liu and Zukin 2007). CP‐AMPARs can be recruited to hippocampal CA1 synapses by Aβ oligomers, potentially impacting synaptic plasticity and memory (Gaidin and Kosenkov 2023; Whitcomb et al. 2015).

Age‐related memory loss can be reversed by enhancing autophagy via increasing dendritic spines and LTP (Glatigny et al. 2019). In this context, autophagy was reported to play a key role in promoting the formation of new memory by modulating structural and functional synaptic plasticity in the hippocampal neurons. Although disrupted synaptic plasticity has been shown in mouse models lacking autophagy, the cellular mechanisms by which it regulates plasticity and behavior, especially in AD, remain elusive (Lieberman and Sulzer 2020). Downregulation of autophagy‐related proteins like *NRBF2* in AD brains is associated with LTP and memory deficits (Lachance et al. 2019). This highlights the connection between LTP, autophagy, and AD, suggesting that enhancing autophagy could restore synaptic function and mitigate cognitive decline.

Multiple studies have shown that synaptic tagging and capture (STC), a mechanism for associative memory, is impaired in AD (Li et al. 2017; Pavon et al. 2024; Raghuraman et al. 2022). Targeting dysregulated signaling pathways has been effective in restoring associative plasticity, with interventions like metaplasticity, G9a/GLP inhibition, Nogo A inhibition, and miRNA‐134‐5p modulation in AD models (Pavon et al. 2024; Singh et al. 2022; Varma et al. 2023) (Baby et al. 2020; Li et al. 2017; Pavon et al. 2024; Raghuraman et al. 2022; Singh et al. 2022). STC is particularly vulnerable to neurodegeneration and aging, often showing impairments even when late‐LTP remains intact, making it a useful marker for early plasticity deficits (Bin Ibrahim et al. 2024; Shetty et al. 2017).

In this study, we investigated the effect of AKG/CaAKG on LTP and its underlying cellular mechanisms in APP/PS1 mice. We show that AKG/CaAKG ameliorates synaptic deficits in the AD model, with more profound effects in female AD mice than male AD mice. CaAKG improves plasticity deficits through an NMDAR‐independent mechanism that depends on LTCC and CP‐AMPARs. mTOR inhibition by rapamycin showed differential effects, as it rescued LTP deficits in AD mice but blocked LTP in hippocampal slices from WT mice. Western blot results show an increase in autophagy markers with CaAKG in APP/PS1 mice. Furthermore, CaAKG facilitated synaptic tagging and capture in AD mice, suggesting its role in associative plasticity. These findings suggest that CaAKG holds promise as a therapeutic strategy for AD by enhancing synaptic plasticity and promoting associative plasticity, particularly through mechanisms that involve LTCC, CP‐AMPARs, and autophagy modulation.

## 2 Materials and Methods

### 2.1 Animals

We used a mouse model of AD called APP/PS1, which expresses a mutated chimeric mouse/human amyloid precursor protein (APP) and the exon‐9 deleted variant of human presenilin 1 (PS1) under the control of a prion promoter element (*APPSwe/PS1dE9*) (Borchelt et al. 1997). Both mutations of APP and presenilin 1 are associated with early‐onset familial AD (Lanoiselée et al. 2017). APP/PS1 transgenic mice aged 4–5 months were used for all experiments. We have previously shown that mice at this age exhibit early pathological features of AD, including impaired LTP, memory deficits, increased amyloid beta (Aβ) plaque burden, microglial activation, and altered expression of genes related to synaptic plasticity and memory (Navakkode et al. 2021). All animals were housed under a 12/12 h light/dark cycle and supplied with food and water ad libitum. A total of 200 hippocampal slices prepared from 51 APP/PS1 and 52 WT mice were used for electrophysiological recordings. For western blotting, slices were collected at the end of the recording and snap frozen. All experimental procedures involving animals were performed in accordance with protocols approved by the Institutional Animal Care and Use Committee (IACUC) of the National University of Singapore.

### 2.2 Hippocampal Slice Preparation and Electrophysiology

For acute hippocampal slice preparation, mice were anesthetized using CO_2_ and decapitated. The brain was immediately removed and transferred to cold (2°C–4°C) artificial cerebrospinal fluid (ACSF). Both the left and right hippocampi were isolated in cold ACSF and subsequently sliced into 400 μm transverse sections with a manual tissue chopper. For field electrophysiology, the slices were quickly transferred to an interface chamber (Scientific System Design, Ontario, Canada) which is maintained at 32°C, continuously perfused with oxygenated ACSF equilibrated with 95% O_2_‐5% CO_2_ (carbogen) (total consumption: 16 L/h) at a flow rate of 0.89 mL/min. Recordings were performed at 32°C, a temperature commonly used to preserve slice viability, maintain metabolic stability, and support stable physiological functions especially during long‐term recordings (Sajikumar et al. 2005). Carbogen (96% O₂ and 4% CO₂) was used to bubble the recording chamber and ACSF to ensure sufficient tissue oxygenation and maintain a stable pH (7.3–7.4). The ACSF contained the following (in mM): 124 NaCl, 4.9 KCl, 1.2 KH_2_PO_4_, 2.0 MgSO_4_, 2.0 CaCl_2_, 24.6 NaHCO_3_, and 10 D‐glucose, equilibrated with 95% O_2_/5% CO_2_ (32 L/h; Li et al. 2017).

**FIGURE 1:**
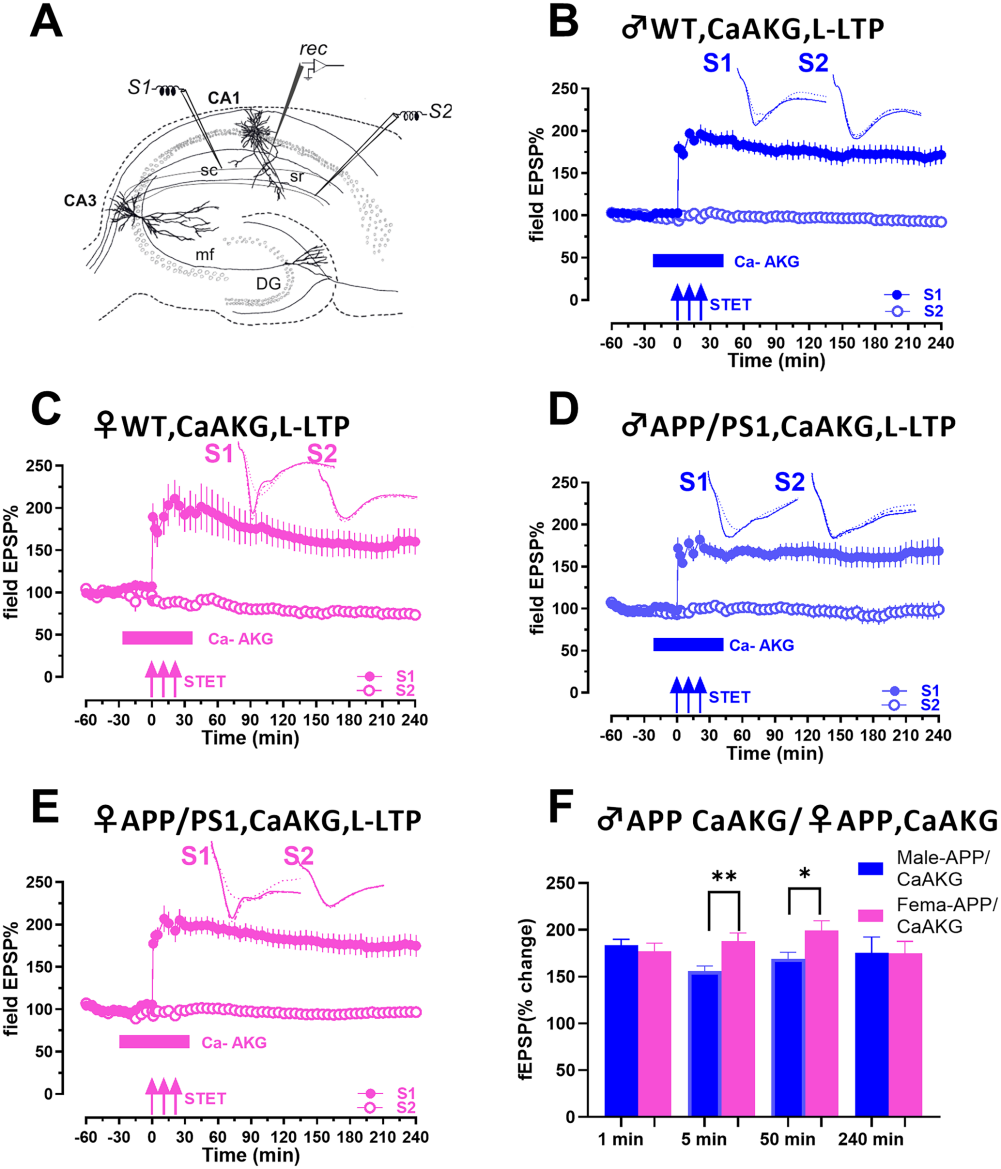
CaAKG ameliorates impaired LTP in APP/PS1 mice with a more profound effect in APP/PS1 females compared to males. (A) Schematic representation of a transverse hippocampal slice with electrodes located in the CA1 area of hippocampus. Recording electrode represented as ‘rec’ is located in CA1 apical dendrites, flanked by two stimulating electrodes S1 and S2 placed in the stratum radiatum (sr) layer to stimulate two independent synaptic inputs to the same neuronal population in the Schaffer collateral (sc) synapses of hippocampus. (B) STET in S1 in presence of CaAKG resulted in a significant potentiation that was maintained for 4 h in male WT mice. CaAKG was bath applied for 60, 30 min before and 30 min after STET. The STET in S1 (blue filled circles) resulted in a significant potentiation that maintained for 4 h, whereas the control potentials in S2 (blue open circles) remained stable throughout the recording (*n* = 9). (C) STET in S1 in female WT mice in the presence of CaAKG also resulted in a potentiation that maintained for 4 h. STET in S1 (pink filled circles) resulted in a long‐lasting LTP for 4 h, whereas the control input S2 (pink open circles) remained stable throughout the time course of recording (*n* = 5). (D) When STET was delivered to S1 (blue filled circles) in male APP/PS1 mice in presence of CaAKG, it resulted in a long lasting LTP. (*n* = 8). (E) STET in S1 (pink filled circles) in presence of CaAKG in female APP/PS1 mice also resulted in a long lasting LTP that persisted for 4 h (*n* = 8). (F) Bar graphs shows comparison of fEPSP values of S1 from male APP/PS1 and female APP/PS1 treated with CaAKG (D, E) at 1, 5, 50‐, and 240‐min post STET. Blue bars represent the values from male APP/PS1 and pink bars represents value from female APP/PS1 mice. Control stimulation of S2 in B, C, D and E showed stable potentials for the recorded time period (blue and pink open circles). Three solid arrows represent the time of induction of late‐LTP by STET. Solid horizontal bars represent the time period of application of CaAKG. Error bars in all graphs indicate ± SEM.

In all electrophysiological recordings, two‐pathway experiments were performed. Two monopolar lacquer‐coated, stainless‐steel electrodes (5 MΩ; AM Systems) were positioned at an adequate distance within the stratum radiatum of the CA1 region for stimulating two independent synaptic inputs S1 and S2 of one neuronal population (Figure 1A), thus evoking field excitatory post synaptic potential (fEPSP) from Schaffer collateral/commissural‐CA1 synapses. Pathway specificity was tested by using a previously described method (Li et al. 2017). For recording the fEPSP (measured as its initial slope function), one electrode (5 MΩ; AM Systems) was placed in the CA1 apical dendritic layer, and signals were amplified by a differential amplifier (model 1700; AM Systems). The signals were digitized by using a CED 1401 analog‐to‐digital converter (Cambridge Electronic Design) and monitored online.

After 3 h incubation, a synaptic I‐O curve (input–output curve) (afferent stimulation vs. fEPSP slope) was generated. Test stimulation intensity was adjusted to elicit a fEPSP slope of 40% of the maximal slope response for both synaptic inputs S1 and S2. Test stimulation comprising four 0.2‐Hz biphasic constant‐current pulses (0.1 ms per polarity) was used at each time point. In all experiments, a stable baseline was recorded for at least 30 min before plasticity induction. To induce late‐LTP, a strong tetanization (STET) protocol, consisting of three high‐frequency stimulations of 100 pulses at 100 Hz (single burst, stimulus duration of 0.2 ms per polarity) with an inter‐train interval of 10 min, was used. To induce early‐LTP, a weak tetanization (WTET) protocol consisting of a single stimulus train of 21 pulses at 100 Hz (stimulus duration of 0.2 ms per polarity) was used.

### 2.3 Pharmacology

AKG was dissolved in water and then in ACSF to a final concentration of 1 mM. CaAKG was first dissolved in 0.1 M HCl and then in ACSF to a 1 mM concentration. AP‐5 or nifedipine (Tocris) was used at a concentration of 50 and 10 μM, respectively (dissolved in ACSF and 0.1% DMSO). We performed preliminary tests to determine an appropriate concentration of Ca‐AKG. We found that 1 mM Ca‐AKG had no significant effect on basal synaptic transmission in hippocampal slices from WT or APP/PS1 mice (data not shown), indicating that this concentration does not interfere with baseline neuronal function. Based on this, we selected 1 mM for all subsequent electrophysiological experiments.

IEM‐1460 (IEM; Tocris), an inhibitor of CP‐AMPARs, was dissolved in water and used at a final concentration of 30 μM (Park et al. 2016). mTOR inhibitor rapamycin (Tocris Bioscience, Bristol, England) was dissolved in DMSO to create a 1 mM stock and was diluted in ACSF to a final concentration of 1 μM. For stocks prepared in DMSO, the final DMSO concentration was kept below 0.1%, a concentration that was shown to not affect basal synaptic responses (Navakkode et al. 2004).

### 2.4 Statistical Analyses

In field electrophysiological recordings, synaptic efficacy was assessed by measuring the slope of the fEPSP (in millivolts per millisecond). All data are presented as mean ± SEM. To evaluate statistical significance within a group, the Wilcoxon signed‐rank test (noted as Wilcox) was employed to compare the mean normalized fEPSPs at specific time points with the baseline fEPSP measured at −15 min. For comparisons between different groups, the Mann–Whitney *U*‐test (noted as *U*‐test) was used. Statistical significance was determined at P < 0.05. Nonparametric tests were chosen because a Gaussian normal distribution could not always be assumed, particularly due to the small sample sizes in each series and the analysis of extended recordings (Li et al. 2017; Navakkode et al. 2007). The variable “*n*” indicates the number of slices used in in vitro electrophysiology or the number of animals in Western blot experiments. For all in vitro and biochemical studies, slices from a minimum of 3–4 biological replicates were included. All graphs and statistical analyses were created using Graph Pad Prism 11.

### 2.5 Western Blotting

After recording, hippocampal slices were carefully taken from the recording chamber, snap frozen in liquid nitrogen, and then stored at −80°C. Proteins were extracted from tissues using Tissue Protein Extraction reagent (T‐PER; Thermo Scientific, Waltham, MA, USA) followed by centrifugation for 5 min at 10,000 rpm at 4°C. The protein concentration was determined using the Bradford assay (Bio‐Rad, Hercules, CA, USA). Suitable amounts of protein were added to the sample buffer and heated at 95°C for 10 min before being separated by SDS‐polyacrylamide gels. Gels were transferred to Nitrocellulose membranes (Bio‐Rad, Hercules, CA, USA) for 2 h at 100 V or 30 V overnight at 4°C. The membranes were blocked with 5% w/v nonfat dry milk in 1X Tris‐buffered saline with Tween 20 (TBST) and then immunoblotted with primary antibodies. The primary antibodies with their concentrations used were as follows: rabbit anti‐LC3 (1:1000, Cell Signaling, Danvers, MA, USA, #3868S) and mouse anti‐tubulin (1:20,000; Sigma‐Aldrich, Darmstadt, Germany, #T6557). The membranes were incubated with respective secondary peroxidase‐conjugated antibodies. SuperSignal West Pico Chemiluminescent Substrate (Thermo Scientific, Waltham, MA, USA) was used to generate signals for imaging. The amounts of all the proteins were quantified by densitometric measurement of Western blots using ImageJ (NIH software). The densitometric values of each blot were normalized to the amounts of tubulin, which served as the loading control.

## 3 Results

### 3.1 CaAKG Ameliorated Hippocampal LTP Deficits in APP/PS1 Mice With Enhanced Effects in Females

We first evaluated the impact of CaAKG on hippocampal synaptic plasticity in male wild‐type (WT) mice (Figure 1B). Application of CaAKG in hippocampal slices from male WT mice led to stable potentiation lasting up to 240 min. Strong tetanisation (STET) in S1 (blue filled circles) resulted in a significant increase in fEPSP values from min 1 (*p =* 0.0039, Wilcoxon test; *p =* < 0.0001, *U* test) post‐ STET and remained significant until 240 min (*p =* 0.0039, Wilcoxon test; *p =* < 0.0001, *U* test) compared to its own baseline (at −15 min) or the corresponding control S2 (blue open circles) (*n* = 9). Next, we tested whether CaAKG shows a sex‐dependent effect in WT mice, so we tested its effects in female WT mice. In female WT mice (Figure 1C) STET in S1 (pink filled circles) in the presence of CaAKG led to an increase in fEPSP values at 1 min (*p =* 0.0312, Wilcoxon test; *p =* 0.0022, *U* test) and it remained stable and significant until 240 min (*p =* 0.0312, Wilcoxon test; *p =* 0.0022, *U* test) compared to its own baseline or the S2 (open pink circles) (*n* = 5).

To investigate whether CaAKG has a protective effect in AD mice, we first tested its effect in male APP/PS1 mice. In male APP/PS1 mice (Figure 1D) CaAKG rescued the impaired LTP. STET in S1 (blue filled circles) led to a significant potentiation that was stable from 1 min (*p =* 0.0156, Wilcoxon test; *p =* 0.0037, *U* test) until 240 min (*p =* 0.0078, Wilcoxon test; *p =* 0.0037, *U* test, (*n* = 8)). In female APP/PS1 mice (Figure 1E), STET in S1 (pink filled circles) in the presence of CaAKG led to a significant potentiation that was stable from 1 min (*p =* 0.0078, Wilcoxon test; *p =* 0.0002, *U* test) until 240 min (*p =* 0.0156, Wilcoxon test; *p =* 0.0030, *U* test (*n* = 8)).

Comparison of fEPSP values between WT male and WT female mice did not show any difference at any time points (comparison of Figure 1B,C). Interestingly, we observed that CaAKG exerted a more profound effect in APP/PS1 females compared to APP/PS1 males (Figure 1F; Figure 1D vs. 1E). Comparison of fEPSP values from input S1 of male and female APP/PS1 treated with CaAKG showed that females showed a higher potentiation compared to males from 5 to 50 min (5 min, *p =* 0.0019, *U* test; 30 min, *p =* 0.0499, *U* test; 50 min, *p =* 0.0379, *U* test).

Comparison between control male APP/PS1 mice with male APP/PS1 mice treated with CaAKG (Figure S1C vs. Figure 1D) shows that the values are significant from 120 min onwards (120 min, *p =* 0.0426, *U* test). In contrast, comparison of control female APP/PS1 mice with female APP/PS1 mice treated with CaAKG (Figure S1D vs. Figure 1E) shows that fEPSP values were statistically significant from 1 min onwards until 240 min (1 min, *p =* 0.0093, *U* test; 60 min, *p =* 0.0006, *U* test; 180 min, *p =* 0.0037, *U* test; 240 min, *p =* 0.0037, *U* test). These comparisons of control male and female APP/PS1 mice with the corresponding CaAKG treated ones clearly strengthen our findings that female APP/PS1 mice have a profound effect with CaAKG compared to male APP/PS1 mice. Control input S2 in all experiments (blue and pink open circles) showed stable fEPSP values throughout the recordings. Overall, our results indicate that CaAKG ameliorated synaptic deficits in APP/PS1 mice, with female AD mice having a more profound effect than male AD mice.

### 3.2 AKG Enhanced Hippocampal LTP in APP/PS1 Mice

**FIGURE 2:**
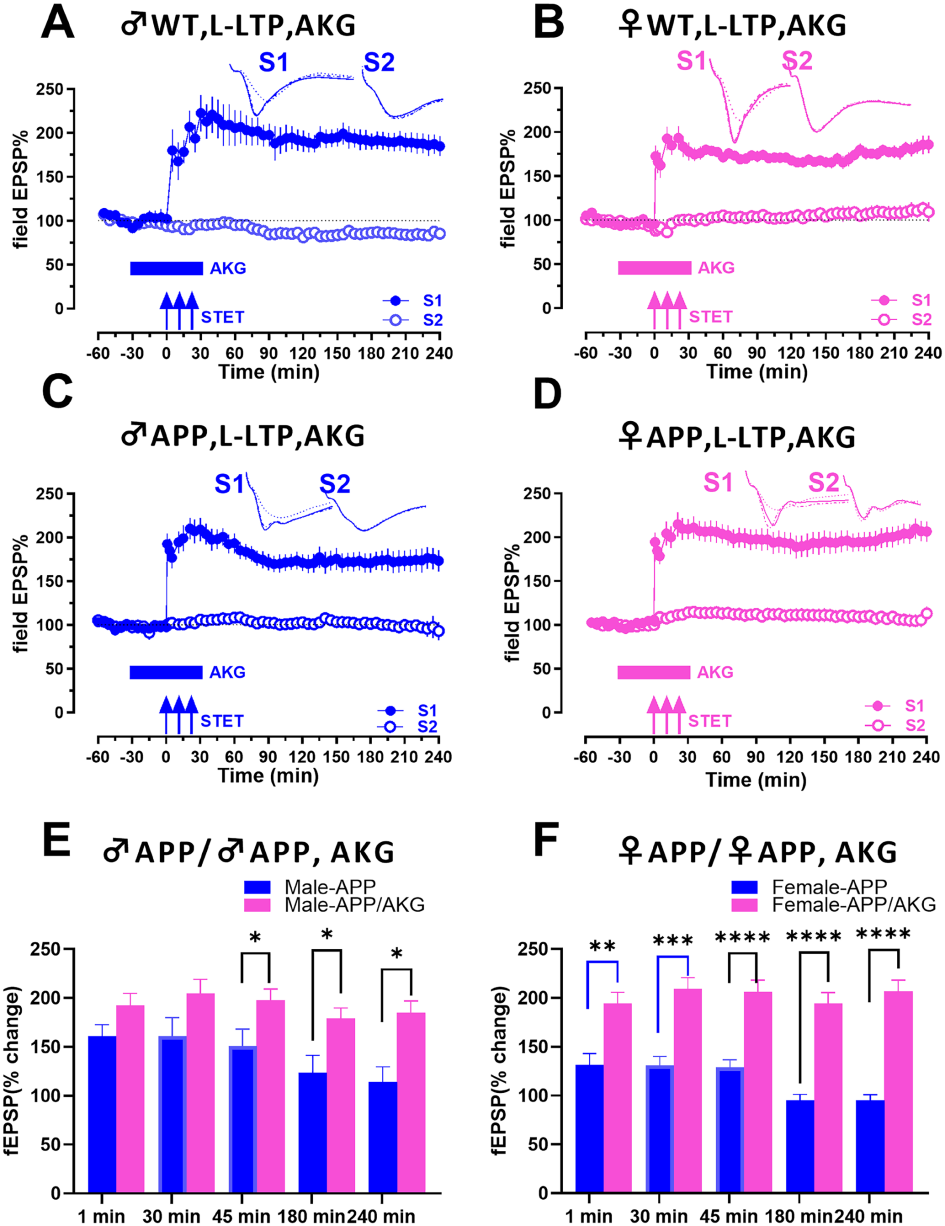
AKG ameliorates impaired LTP in APP/PS1 mice with an enhanced effect in APP/PS1 females. (A) Induction of late‐LTP by STET in synaptic input S1 in male WT mice in presence of AKG resulted in a potentiation that remained stable for 240 min (blue filled circles, *n* = 7). Although the control input S2 (blue open circles) remained stable throughout the recording (blue open circles). (B) Treatment of hippocampal slices with AKG resulted in late‐LTP that was stable for 4 h in S1 (pink filled circles) in female WT mice. Although the control input S2 (pink open circles) remained stable throughout the time course of recording (*n* = 5). (C) STET was delivered to hippocampal slices from male APP/PS1 mice in presence of AKG, which resulted in a long lasting LTP. (*n* = 8). (D) When STET was delivered to hippocampal slices from female APP/PS1 mice in presence of AKG, it also resulted in a long lasting LTP (*n* = 10). Control stimulation of S2 in both C and D showed stable potentials for the recorded time period of 4 h (blue and pink open circles). (E) Bar graphs shows comparison of fEPSP values of S1 from control male APP/PS1 and male APP/PS1 treated with AKG (Figure S1C; C) at 1, 30, 45, 180‐ and 240‐min post STET. (F) Bar graphs shows comparison of fEPSP values of S1 from control female APP/PS1 and female APP/PS1 treated with AKG (Figure S1D; D) at 1, 30, 45, 180‐ and 240‐min post STET. Blue bars represent the values from male APP/PS1 and pink bars represents the value from females. Three solid arrows represent the time of induction of L‐LTP by STET for the induction of late‐LTP. Horizontal solid bars represent the time period of application of AKG. Error bars in all graphs indicate ± SEM.

Next, we investigated whether pure AKG has any effect on LTP in APP/PS1 mice. Since CaAKG is a source of calcium (Ca^2+^) in addition to AKG and moreover Ca^2+^ ions play a significant role in cellular signaling, we used pure AKG as a control to see if AKG alone has a differential effect. We first examined the role of AKG in male WT hippocampal slices (Figure 2A). Bath application of AKG during the STET in hippocampal slices did not induce a significant effect. STET in S1 (blue filled circles) led to a potentiation that was statistically significant from 1 min (1 min, *p =* 0.0010, Wilcoxon test; *p =* 0.0007, *U* test) until 240 min (240 min, *p =* 0.0010, Wilcoxon test; *p =* 0.0007, *U* test (*n* = 7)). *p* values were not significant at any time points between control WT and those treated with AKG *p* < 0.05. When WT female (Figure 2B) hippocampal slices were bath applied with AKG during LTP, a long‐lasting, input‐specific potentiation was observed in S1 (pink filled circles), which was statistically significant from 1 min (1 min, *p =* 0.0312, Wilcoxon test; *p =* 0.0022, *U* test) until 240 min (240 min, *p* = 0.0312, Wilcoxon test; *p =* 0.0022, *U* test) (*n* = 5). No significant difference was found between female WT controls and those treated with AKG *p* < 0.05. To test whether AKG has any effect on AD mice, we used male APP/PS1 mice first. In male APP/PS1 (Figure 2C) mice, the application of AKG rescued the impaired plasticity. Induction of LTP with STET in S1 (blue filled circles) led to an increase in fEPSP values that remained stable from 1 min (1 min, *p =* 0.0156, Wilcoxon test; *p* = < 0.0001, *U* test) until 240 min (240 min, *p =* 0.0312, Wilcoxon test; *p =* 0.0238, *U* test) (*n* = 8). To study if AKG induces any sex‐dependent effects, we studied its effects in female AD mice. In female APP/PS1 mice (Figure 2D) also STET in S1 (pink filled circles), in the presence of AKG led to an increase in fEPSP values from 1 min (1 min, *p =* 0.0020, Wilcoxon test; *p =* < 0.0001, *U* test) until 240 min (240 min, *p =* 0.0020, Wilcoxon test; *p =* < 0.0001, *U* test (*n* = 10)).

No significant difference was found between WT males and females when compared to those treated with AKG (Figure 2A vs. 2B). fEPSP values were compared between male AD and female AD mice treated with AKG (Figure 2C,D). No significant changes were observed at any time points from 1 min until 240 min when comparing S1 between male and female APP/PS1 mice treated with AKG (1 min, *p =* > 0.9999, *U* test; 60 min, *p =* 0.5148, *U* test; 180 min, *p =* 0.1728, *U* test; 240 min, *p =* 0.0934, *U* test). But comparison of control male APP/PS1 mice with the male APP/PS1 treated with AKG (Figure 2E; Figure S1C vs. Figure 2C) showed statistically significant differences in fEPSP values from 45 min onward (45 min, *p =* 0.0007, *U* test). In contrast, comparison of control female APP/PS1 mice with female APP/PS1 mice treated with AKG (Figure 2F; Figure S1D vs. Figure 2D) showed fEPSP values that were statistically significant from 1 min onwards until 240 min (1 min, *p =* 0.0046, *U* test; 60 min, *p =* 0.000, *U* test; 180 min, *p =* 0.0001, *U* test; 240 min, *p =* 0.0001, *U* test). In all recordings, the control input S2, fEPSP values were stable throughout the recording (blue and pink open circles).

These results indicate that AKG restores input‐specific synaptic potentiation in hippocampal slices from APP/PS1 mice, with a potentially stronger restorative effect observed in female AD mice, but these differences were observed only when compared with its control untreated animals. Furthermore, a comparison between AKG and CaAKG‐treated slices revealed no differences *p* < 0.05, confirming that the Ca^2+^ in CaAKG did not have any additional effects in our studies.

### 3.3 CaAKG Mediated an NMDAR‐Independent but L‐Type Calcium Channel Dependent Signaling

**FIGURE 3:**
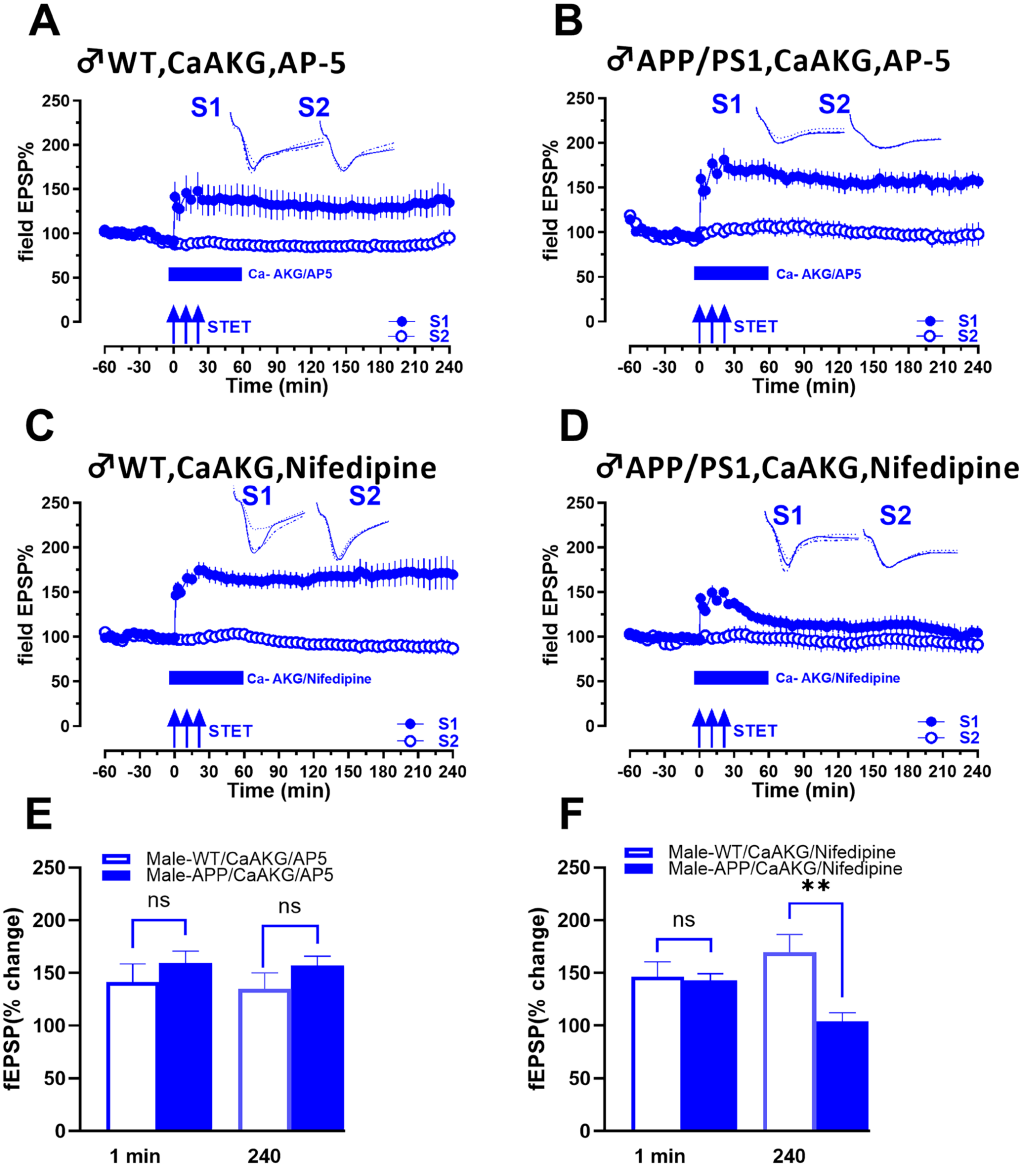
CaAKG induces an NMDAR‐independent and LTCC dependent mechanism. (A) In male WT mice, treatment of hippocampal slices with CaAKG and AP5 in WT mice resulted in late‐LTP in S1 (blue filled circles), whereas the control input S2 remained stable throughout the recording (open blue circles) (*n* = 7). (B) In APP/PS1 mice, treatment of hippocampal slices with CaAKG and AP5 resulted in late‐LTP in S1 (blue filled circles), similar to WT LTP (*n* = 8). (C) Induction of late‐LTP by STET in synaptic input S1 (blue filled circles) in presence of CaAKG and an LTCC blocker, nifedipine in WT mice resulted in a potentiation that remained stable for 240 min (blue filled circles) (*n* = 7). (D) However, in APP/PS1 mice, co‐application of CaAKG and nifedipine during the induction of late‐LTP by STET in synaptic input S1 resulted only in early‐LTP in S1 (blue filled circles). Control potentials from S2 (open blue circles) in all experiments remained stable during the recording period (*n* = 8). (E) Bar graph compares male WT mice treated with CaAKG and AP‐5 with male APP/PS1 mice treated with CaAKG and AP‐5 (A vs. B). (F) Bar graph compares male WT mice treated with CaAKG and Nifedipine with male APP/PS1 mice treated with CaAKG and Nifedipine (C vs. D). Error bars indicate ± SEM.

Multiple studies have shown that inhibition of NMDARs completely blocks STET induced LTP, suggesting that NMDARs are critical for LTP (Dozmorov et al. 2006; Navakkode et al. 2007; Pananceau and Gustafsson 1997; Vickers et al. 2005). To test whether CaAKG‐facilitated LTP is NMDAR‐dependent, we blocked NMDARs with AP‐5 in both male WT and male APP/PS1 mice treated with CaAKG. When STET was applied in the presence of CaAKG and AP‐5 in male WT mice (Figure 3A), significant potentiation of fEPSP values was observed in S1 (blue filled circles) from 1 min (1 min, *p =* 0.0312, Wilcoxon test; *p =* 0.0002, *U* test) to 240 min (240 min, *p =* 0.0312, Wilcoxon test; *p =* 0.0048, *U* test) (*n* = 7). In APP/PS1 mice, the co‐application of CaAKG and AP‐5 also showed statistically significant LTP for 4 h (Figure 3B). STET in S1 (blue filled circles) induced potentiation that was statistically significant from 1 min (1 min, *p =* 0.0312, Wilcoxon test; *p =* 0.0043, *U* test) to 240 min (240 min, *p =* 0.0312, Wilcoxon test; *p =* 0.0152, *U* test (*n* = 8)). These results clearly suggest that CaAKG ameliorated LTP through an NMDAR‐independent mechanism. Ca^2+^ entry through LTCCs could serve as an alternative for NMDAR‐independent LTP; therefore, we tested whether CaAKG‐mediated LTP is dependent on LTCCs. An LTCC blocker, nifedipine, applied along with CaAKG during STET in S1 (blue filled circles) in male WT hippocampal slices did not affect late‐LTP (*n* = 7) (Figure 3C). However, in APP/PS1 mice (Figure 3D) application of CaAKG and nifedipine impaired late‐LTP, showing that in APP/PS1 mice, CaAKG mediated its effects via LTCCs. STET in S1 (blue filled circles) led to an increase in fEPSP values that were statistically significant from 1 min (1 min, *p =* 0.0039, Wilcoxon test; *p =* < 0.0001, *U* test) to 65 min (65 min *p =* 0.0273, Wilcoxon test) compared to its baseline, and from 70 min onwards it decayed to baseline (70 min *p =* 0.0547, Wilcoxon test) (*n* = 8). It remained statistically significant until 40 min (40 min, *p =* 0.0188, *U* test) when compared to S2 and decayed to baseline from 45 min (45 min, *p =* 0.0508, *U* test). Control potentials in S2 remained stable throughout the recording period in experiments (blue open circles). The bar graph (Figure 3E) compares male WT mice treated with CaAKG/AP‐5 and male APP/PS1 mice treated with CaAKG and AP‐5 at 1 min and 240 min (Figure 3A vs. 3B). The values show no significant difference between the groups at either time point (1 min, *p =* 0.1520, *U* test, 240 min, *p =* 0.0721, *U* test). The bar graph (Figure 3F) compares male WT treated with CaAKG and nifedipine and male APP/PS1 mice following CaAKG and nifedipine treatment (Figure 3C vs. 3D). There is no significant difference at 1 min; however, a significant difference is observed at 240 min (1 min, *p =* 0.7577, *U* test, 240 min, *p =* 0.0021, *U* test). Our findings indicate that CaAKG reinstates LTP in APP/PS1 mice through an NMDAR‐independent and LTCC‐dependent mechanism.

### 3.4 Calcium Permeable AMPA Receptors Are Involved in the Effect of CaAKG in APP/PS1 Mice

**FIGURE 4:**
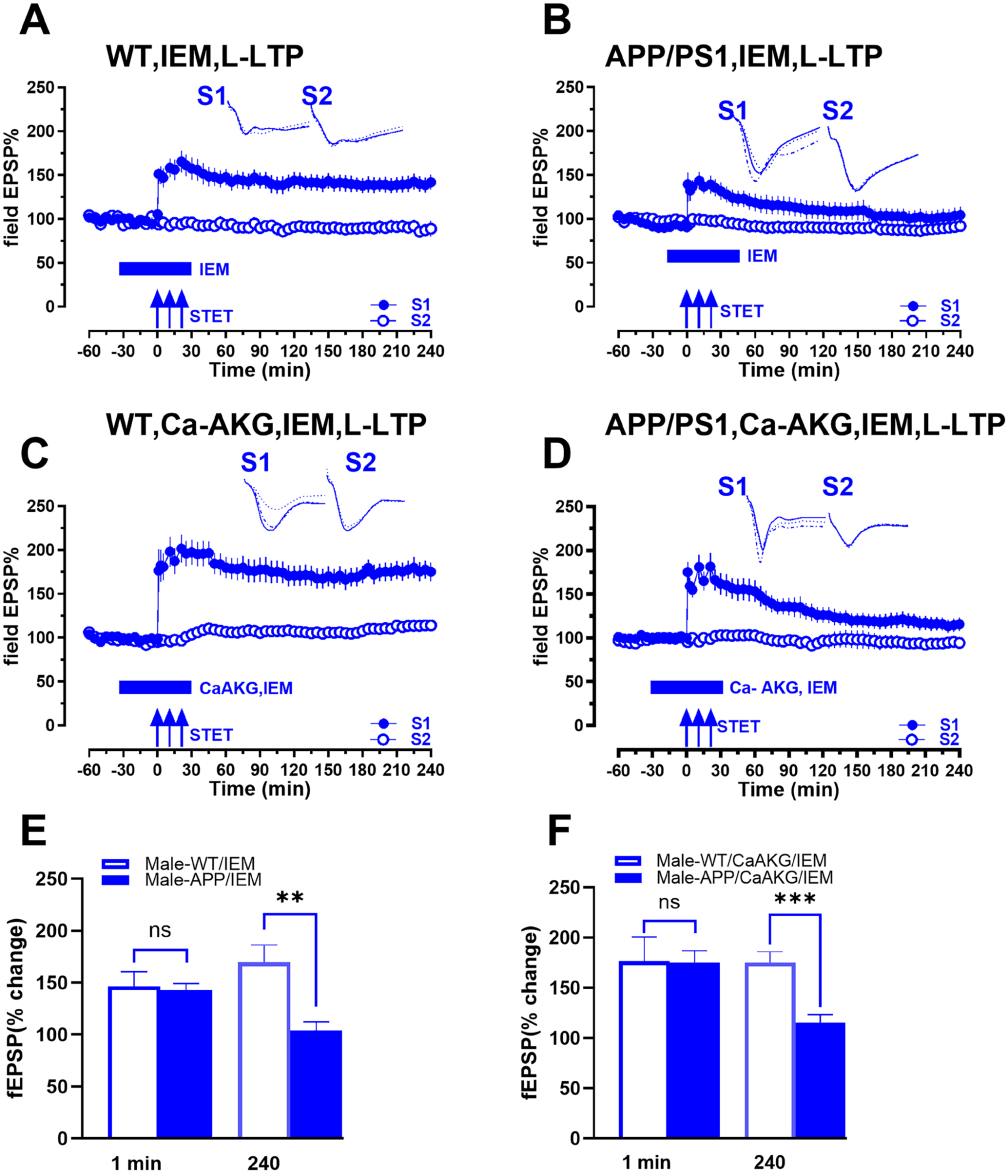
Calcium‐permeable AMPARs are involved in the effect of CaAKG. (A) Treatment of hippocampal slices with IEM during STET in synaptic input S1 (blue filled circles) from WT mice resulted in late‐LTP that was maintained until the end of the recording (*n* = 7). (B) In APP/PS1 mice STET in synaptic input S1 (blue filled circles) in presence of IEM resulted only in an early‐LTP that gradually decayed to the baseline potentials (*n* = 7). (C) Co‐treatment of WT hippocampal slices with CaAKG and IEM also resulted in late‐LTP in S1 (blue filled circles), when STET was delivered (*n* = 7). (D) Although in APP/PS1 mice co‐treatment with CaAKG and IEM during STET in synaptic input S1 (blue filled circles) resulted only in an early‐LTP that gradually decayed to the baseline potentials (*n* = 9). Control potentials from S2 (blue open circles) remained stable during the recording period in all experiments. (E) Bar graph compares male WT mice treated with IEM with male APP/PS1 mice treated with IEM (A vs. B). (F) Bar graph compares male WT mice treated with CaAKG and IEM with male APP/PS1 mice treated with CaAKG and IEM (C vs. D).

The Ca^2+^ conductance of CP‐AMPARs may have both physiological and pathological roles. Thus, we were interested in evaluating whether the effects of CaAKG involve the action of CP‐AMPARs and whether this is pathological or neuroprotective. First, we examined if inhibition of CP‐AMPAR affects late‐LTP in male WT mice (Figure 4A). IEM, an inhibitor of CP‐AMPARs, was bath applied for 60 min, 30 min before and 30 min after STET in S1 (blue filled circles). IEM had no effect on the induction and maintenance of LTP in WT slices. In the presence of IEM, STET led to an increase in fEPSP values that was statistically significant from 1 min (1 min, *p =* 0.0312, Wilcoxon test; *p =* 0.0022, *U* test (*n* = 7)) until 240 min (240 min, *p =* 0.0312, Wilcoxon test; *p =* 0.0087, *U* test). Next, we investigated the role of CP‐AMPARs in APP/PS1 mice (Figure 4B) by repeating the same experiment in male APP/PS1 mice. STET in S1 (blue filled circles) in the presence of IEM led to a significant potentiation at 1 min (1 min, *p =* 0.0234, Wilcoxon test; *p =* 0.0104, *U* test), which remained stable until 50 min when compared to its own baseline (*p =* 0.0391, Wilcoxon test), and 45 min when compared to control S2 (45 min *p =* 0.0281, *U* test (*n* = 7)). Potentiation decayed to baseline from 55 min (*p =* 0.0547, Wilcoxon test) and 50 min (50 min, *p =* 0.0104, *U* test). Next, CaAKG was co‐applied with IEM in WT hippocampal slices (Figure 4C). STET in S1 in the presence of CaAKG and IEM had no additional effect on WT slices. STET led to a potentiation in S1 (blue filled circles) that was statistically significant from 1 min (1 min, *p =* 0.0156, Wilcoxon test; *p =* 0.0082, *U* test) until 240 min (240 min, *p =* 0.0156, Wilcoxon test; *p =* 0.0012, *U* test (*n* = 7)). In contrast, in APP/PS1 mice, co‐application of IEM and CaAKG prevented the expression of persistent LTP by CaAKG (Figure 4D). This indicates that inhibition of CP‐AMPAR by IEM prevented the rescue of LTP by CaAKG in male APP/PS1 mice. STET in S1 (blue filled circles) led to a potentiation that was statistically significant from 1 min (1 min, *p =* 0.0039, Wilcoxon test; *p =* < 0.0001, *U* test) until 135 min (135 min *p =* 0.0039, Wilcoxon test) compared to its own baseline, but from 140 min onwards it decayed to baseline (140 min *p =* 0.0547, Wilcoxon test). It remained statistically significant until 125 min (125 min, *p =* 0.0244, *U* test (*n* = 9)) when compared to S2 and decayed to baseline from 130 min (130 min, *p =* 0.0503, *U* test). Control potentials remained stable throughout the recording period in all experiments (open blue circles). The bar graph (Figure 4E) compares male WT mice treated with IEM and male APP/PS1 mice treated with IEM (Figure 4A vs. 4B) at 1 min and 240 min. The values show no significant difference between the groups at 1 min (1 min, *p =* 0.1520, *U* test) while it shows a significant difference at 240 min (240 min, *p =* 0.0021, *U* test). The bar graph (Figure 4F) compares male WT treated with CaAKG and IEM and male APP/PS1 mice following CaAKG and IEM treatment (Figure 4C vs. 4D). There is no significant difference at 1 min; however, a significant difference is observed at 240 min (1 min, *p =* 0.6807, *U* test, 240 min, *p =* 0.0007, *U* test). Our results suggest that IEM blocks late‐LTP in APP/PS1 mice and that CaAKG does not rescue this impairment.

### 3.5 Rapamycin Rescues Synaptic Deficits in APP/PS1 Mice

A recent study by Tumurbaatar showed that preserved autophagy is a key protective mechanism that maintains cognitive integrity in non‐demented AD pathology (Tumurbaatar et al. 2023). Activation of autophagy is known to rescue synaptic and cognitive deficits in both old and fragile X mice (Glatigny et al. 2019; Yan et al. 2018). This prompted us to check whether increasing autophagy could play a role in improving synaptic deficits in APP/PS1 mice.

**FIGURE 5:**
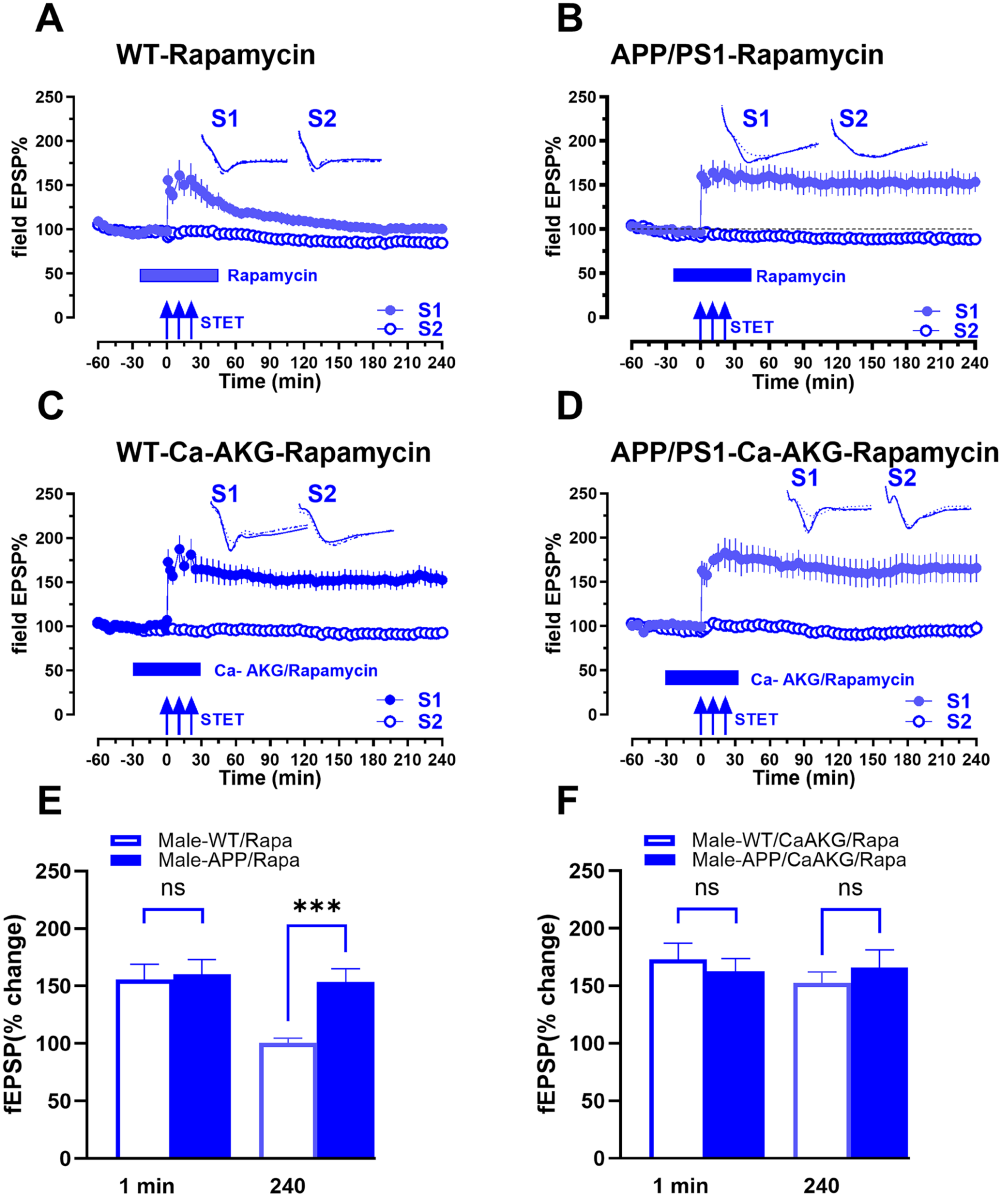
Effects of inhibition of mTOR on late‐LTP in WT and APP/PS1 mice and the effects of CaAKG. (A) The STET in S1 (blue filled circles) resulted in an early‐LTP that gradually decayed to the baseline potentials in WT mice hippocampal slices treated with rapamycin (*n* = 8). (B) The STET in S1 (blue filled circles) resulted in late‐LTP that was maintained until the end of the recording in APP/PS1 mice hippocampal slices treated with rapamycin (*n* = 7). (C) The STET in S1 (blue filled circles) resulted in a significant potentiation that got maintained for 4 h in WT mice hippocampal slices co‐treated with rapamycin and CaAKG (*n* = 6). (D) STET in S1 (blue filled circles) in APP/PS1 mice also resulted in a significant potentiation that was maintained for 4 h in hippocampal slices co‐treated with rapamycin and CaAKG (*n* = 7). Control inputs S2 maintained a stable potentiation throughout the time of recording (blue open circles). (E) Bar graph compares male WT mice treated with rapamycin with male APP/PS1 mice treated with rapamycin (A vs. B). (F) Bar graph compares male WT mice treated with CaAKG and rapamycin with male APP/PS1 mice treated with CaAKG and rapamycin (C vs. D).

Rapamycin is known to upregulate autophagy by inhibiting mammalian target of rapamycin (mTOR), a key regulator of translation initiation and long‐term synaptic plasticity. We first examined the effects of mTOR inhibition by rapamycin on late‐LTP in the CA1 area of the hippocampus from male WT mice (Figure 5A). Rapamycin was applied for 1 h during the tetanisation, from 30 min before to 30 min after the induction of late‐LTP by STET in S1 (blue filled circles). fEPSP values were statistically significant from min 1 (Wilcox test, *p =* 0.0078, *U*‐test, *p* = < 0.0001) until 75 min (Wilcox test, *p =* 0.0078 (*n* = 8)), before declining to the baseline potentials. Next, we studied the effect of mTOR inhibition on APP/PS1 mice (Figure 5B). Application of rapamycin, 30 min before and 30 min after STET in S1 (blue filled circles) rescued LTP in APP/PS1 mice. fEPSP values were statistically significant from 1 min (Wilcox test, *p =* 0.0312, *U*‐test, *p* = 0.0013) after STET until 240 min (Wilcox test, *p =* 0.0312, *U*‐test, *p* = 0.0007 (*n* = 7)). This indicates that enhancing autophagy has the potential to ameliorate synaptic deficits in AD mice, whereas in WT a balance of protein synthesis and degradation has to be maintained to facilitate long‐term synaptic plasticity (Fonseca et al. 2006).

Next to study if application of CaAKG has any impact on rapamycin‐treated slices in WT, rapamycin was coapplied with CaAKG for 1 h during late‐LTP. fEPSP values showed significant potentiation from 1 min (Wilcox test, *p =* 0.1250, *U*‐test, *p* = 0.0286) until 240 min (Wilcox test, *p =* 0.1250, *U*‐test, *p* = 0.0286) (*n* = 6) (Figure 5C). In APP/PS1 mice, induction of late‐LTP by STET in S1 (blue filled circles) in the presence of both rapamycin and CaAKG led to a statistically significant potentiation from 1 min (Wilcox test, *p =* 0.0156, *U*‐test, *p* = 0.0002) until 240 min (Wilcox test, *p =* 0.0312, *U*‐test, *p* = 0.0033 (*n* = 7) (Figure 5D)). Comparison of fEPSP values of S1 (blue filled circles) between APP/PS1 mice treated with rapamycin alone and those co‐treated with CaAKG (Figure 5B,D) showed no significant differences at any time points from 1 min until 240 min (*p* > 0.05). The control synaptic inputs S2 in both WT mice and APP/PS1 in all experiments remained stable throughout the entire recording period (blue open circles). The bar graph (Figure 5E) compares male WT mice treated with rapamycin and male APP/PS1 mice treated with rapamycin (Figure 5A vs. 5B) at 1 and 240 min. The values show no significant difference between the groups at 1 min (1 min, *p =* 0.6126, *U* test) while they show a significant difference at 240 min (240 min, *p =* 0.0003, *U* test). The bar graph (Figure 5F) compares male WT treated with CaAKG and rapamycin and male APP/PS1 mice following CaAKG and rapamycin treatment (Figure 5C vs. 5D). There is no significant difference at either 1 or 240 min (1 min, *p =* 0.4219, *U* test, 240 min, *p =* 0.6020, *U* test). These findings indicate that in WT mice, rapamycin blocked the maintenance of LTP while in APP/PS1 mice, rapamycin rescued the impaired LTP. With CaAKG, LTP remained intact in both WT and APP/PS1 mice. In APP/PS1 mice, CaAKG may act in synergy with rapamycin to enhance the impaired LTP .

### 3.6 CaAKG Enhances Expression of Autophagy Markers in APP/PS1 Mice

**FIGURE 6:**
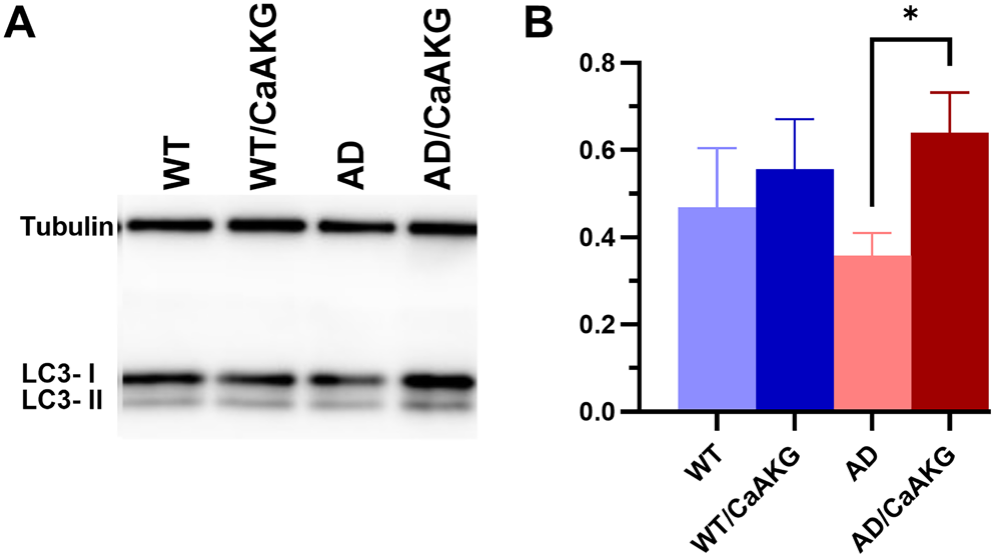
Effects of CaAKG in the expression levels of LC3II in WT and APP/PS1 mice. (A) Western blot analysis of LC3 expression in hippocampal slices from wild‐type (WT), CaAKG‐treated WT (WT/CaAKG), APP/PS1 (AD), and CaAKG‐treated APP/PS1 (AD/CaAKG) mice. LC3‐II levels were markedly increased in APP/PS1 mice treated with CaAKG (dark red bars) compared to control APP/PS1 group (light red bars). Tubulin was used as a loading control. (B) Quantification of LC3‐II levels normalized to tubulin. CaAKG treatment increased LC3‐II levels in APP/PS1 mice (dark red bars) compared to untreated APP/PS1 (*p* = 0.0152, *U*‐test, *n* = 5). Data are presented as mean ± SEM. Asterisk indicates statistically significant difference (*p* < 0.05).

To evaluate the impact of CaAKG on autophagy, we assessed levels of microtubule‐associated protein 1 light chain 3 isoform II (LC3‐II), a widely accepted marker of autophagosome formation. Western blot analysis was performed on hippocampal tissue from both APP/PS1 and wild‐type (WT) mice, with and without CaAKG treatment. Our results revealed a significant reduction in LC3‐II levels in untreated APP/PS1 mice (AD, light red bar) compared to CaAKG treated APP/PS1 slices (AD/CaAKG, dark red bar) (*p* = 0.0152, *U*‐test, *n* = 5), indicating impaired autophagic activity in the APP/PS1 mice and that CaAKG increases autophagy (Figure 6A,B). Although treatment with CaAKG did not elevate LC3‐II expression in WT slices treated with CaAKG slices (WT/CaAKG, dark blue bar) compared to their untreated WT (WT, light blue bar) (*p* = 0.6991, *U*‐test, *n* = 5). These findings suggest that CaAKG selectively enhances autophagic activity in APP/PS1 mice, which may account for the enhanced synaptic activity in AD mice.

### 3.7 CaAKG Facilitates Associative Plasticity via Synaptic Tagging and Capture

**FIGURE 7:**
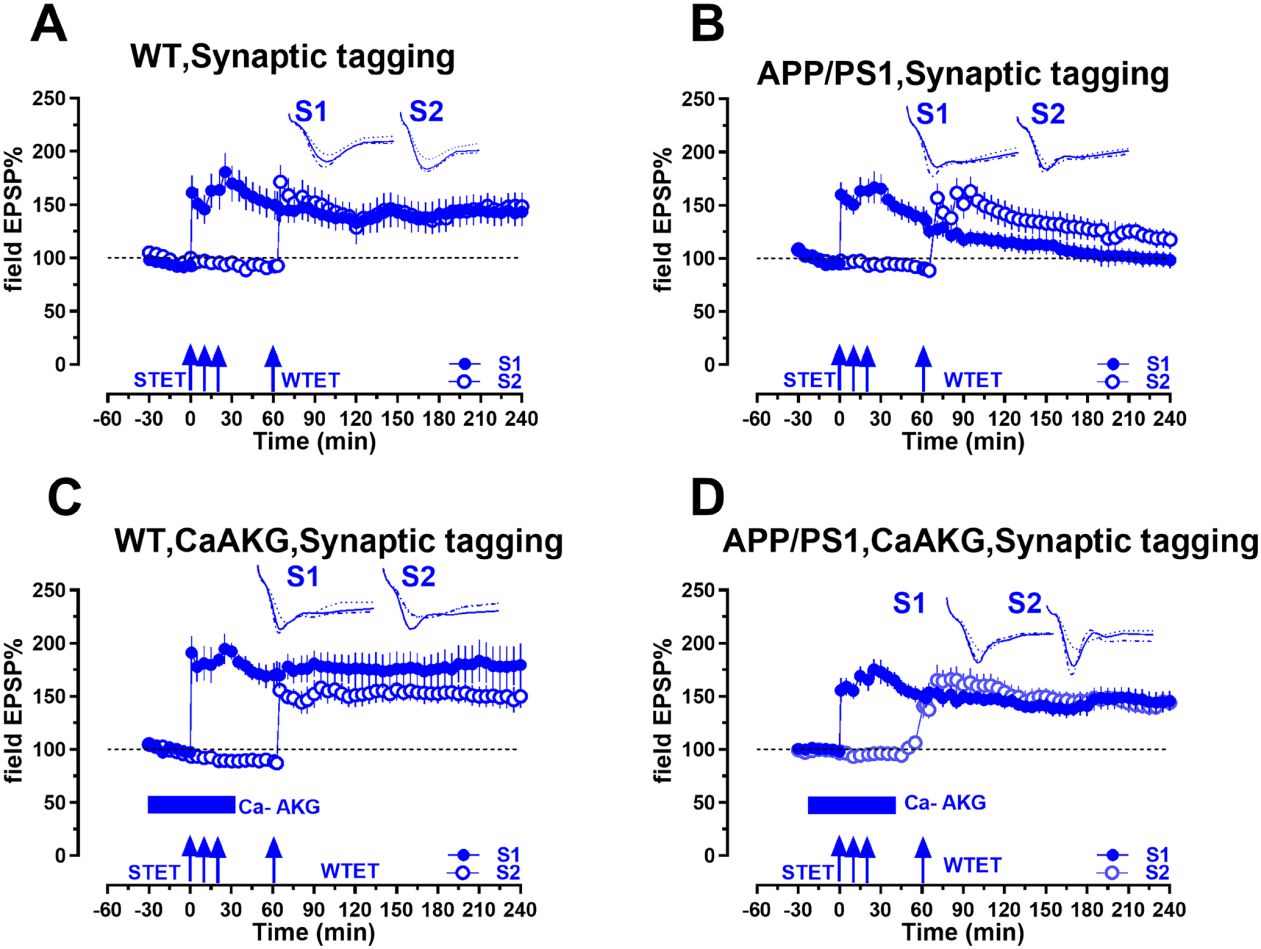
CaAKG mediated Late‐LTP at CA1 synapses engages in synaptic tagging and capture. (A) Synaptic tagging and capture (STC) was tested using ‘strong before weak’ (SBW) paradigm. Induction of early‐LTP in S2 (blue open circles) by WTET, 60 min after the late‐LTP induction in S1 (blue filled circles) by STET resulted in late‐LTP in both the synaptic inputs, thereby expressing STC (*n* = 5). (B) The same paradigm when used in APP/PS1 mice failed to express late‐LTP in both the inputs S1 and S2 (*n* = 7). (C) The same experiment was repeated as in A, but WTET in S2 (blue filled circles) in presence of CaAKG in WT mice. Application of CaAKG did not affect the STC in WT mice (*n* = 6). (D) Application of CaAKG during WTET in S2 (open blue circles) restored the STC in APP/PS1 mice. Here STET in S1 (blue filled circles) that normally leads to early‐LTP was transformed into late‐LTP by STC (*n* = 11).

Synaptic tagging and capture (STC) is impaired in both AD mice models and slices treated with Aβ (1–42) oligomers (Krishna et al. 2016; Li et al. 2017; Pavon et al. 2024; Sharma et al. 2017). Since CaAKG enhanced impaired LTP in AD mice, we set out to study if CaAKG can restore associative plasticity in APP/PS1 mice. To study the effect of CaAKG on STC, we employed the “strong before weak” paradigm, in which STET was delivered to synaptic input S1 prior to WTET in S2 with an interval of 60 min in WT mice (Figure 7A). After a stable baseline was obtained in the inputs S1 and S2, a STET was delivered to S1 (blue filled circles). One hour later, an early LTP which typically decays to baseline within 1–3 h‐ was induced in synaptic input S2 (blue open circles) by a weak tetanization (WTET). STET in S1 resulted in a statistically significant potentiation from 1 min (Wilcox test, *p =* 0.0005) until 240 min (Wilcox test, *p =* 0.0188 (*n* = 5)). After delivery of WTET, S2 expressed an immediate increase in fEPSP that was statistically significant from 1 min until the end of the recording (1 min, Wilcox test, *p =* 0.0307, 240 min, Wilcox test, *p =* 0.0171).

We repeated these experiments in APP/PS1 mice (Figure 7B). In both S1 and S2, the fEPSP values declined to baseline levels. STET in S1 (blue filled circles) induced a potentiation that was statistically significant from 1 min until 90 min (1 min, Wilcox test, *p =* 0.0156, 90 min, Wilcox test, *p =* 0.0469), but from 95 min onwards it was insignificant when compared to its own baseline until 240 min (95 min, Wilcox test, *p =* 0.0781, 240 min, Wilcox test, *p =* 0.9375). WTET in S2 (blue open circles) led to potentiation that remained significant from 1 min until 125 min (1 min, Wilcox test, *p =* 0.0156, 125 min, Wilcox test, *p =* 0.0469), but from 130 min onward, it was insignificant when compared to its own baseline until 240 min (95 min, Wilcox test, *p =* 0.0625, 240 min, Wilcox test, *p =* 0.15625) (*n* = 7).

To study the effects of CaAKG in mediating associative plasticity, we studied its effect in hippocampal slices from WT mice (Figure 7C). A stable baseline was recorded for 30 min after which STET was delivered to S1 (blue filled circles). Thirty minutes later, CaAKG was bath applied for 60 min. Sixty minutes after the STET in S1, a WTET was delivered in S2 (blue open circles), in the presence of CaAKG. WTET in S2 resulted in a long‐lasting LTP by STC. STET in S1 led to statistically significant results from 1 min until 240 min (1 min, Wilcox test, *p =* 0.0312, 240 min, Wilcox test, *p =* 0.0312). In S2, fEPSP values showed significant potentiation from 1 min until 240 min compared to its own baseline (1 min, Wilcox test, *p =* 0.0312; 240 min, Wilcox test, *p =* 0.0312) (*n* = 5).

Next, we examined whether CaAKG can restore long‐term associative plasticity in hippocampal CA1 of APP/PS1 mice. We conducted similar experiments as in Figure 7C in APP/PS1 mice (Figure 7D). A long lasting LTP was observed in both S1 and S2. STET was induced in S1, followed by WTET in S2 in presence of CaAKG. WTET in presence of CaAKG expressed late‐LTP, whereas STET in S1, which normally leads to a short lasting LTP (impaired LTP in APP/PS1 mice), was converted to late‐LTP by STC. fEPSP values were statistically significant at all‐time points compared to its own baseline (1 min, Wilcox test, *p =* 0.0039, 240 min, Wilcox test, *p =* 0.0078). WTET in S2 in the presence of CaAKG converted early‐LTP into late‐LTP. Similar to S1, fEPSP values were significant at all‐time points in S2 from 61 min (1 min after WTET to 240 min) (1 min, Wilcox test, *p =* 0.0156, 240 min, Wilcox test, *p =* 0.0078 (*n* = 9)). Our experiments show that CaAKG can not only facilitate, long‐ term plasticity but also promote associative plasticity in APP/PS1 mice.

## 4 Discussion

Over the past decade, advances in medicine have led to an increase in human lifespan, resulting in a parallel rise in the prevalence of age‐related neurodegenerative disorders such as AD. Although extensive efforts have been directed towards the development of therapeutic interventions to delay onset, prevent progression, or potentially cure AD, none have proven successful. Aging is one of the leading risk factors for AD, and many age‐related biological changes contribute to neurodegeneration in AD. Recently, studies have shown that targeting senescent cells can improve AD symptoms, suggesting that antiaging strategies could offer a promising alternative for AD treatment (Chu et al. 2022; Ho et al. 2010; Soo et al. 2020).

Here, we show that both AKG and calcium alpha‐ketoglutarate (CaAKG) ameliorate synaptic plasticity deficits in the CA1 region of the hippocampus in APP/PS1 mice. Interestingly, the rescue effect was more pronounced in female AD mice, which aligns with our previous findings that CaAKG extends lifespan more significantly in females. The sex differences in the effects of AKG/CaAKG in AD could be due to various factors, including gonadal hormones, especially estrogen, which has a neuroprotective role in the hippocampus by modulating synaptic plasticity, reducing inflammation, and enhancing learning and memory (Brann et al. 2007). We previously showed that female AD mice exhibit faster cognitive and synaptic plasticity deterioration, along with a greater amyloid burden and neuroinflammation compared to males (Navakkode et al. 2021). This could explain the heightened sensitivity of AKG and CaAKG in females. This is supported by the study showing that CaAKG improves longevity in females by decreasing the inflammation (Asadi Shahmirzadi et al. 2020). Shahmirzadi has shown earlier that AKG exerts anti‐inflammatory effects by modulating the senescence‐associated secretory phenotype (SASP), thereby reducing the secretion of pro‐inflammatory cytokines without affecting the formation of senescent cells. It enhances IL‐10 production from T cells, a key anti‐inflammatory cytokine that plays a critical role in suppressing chronic inflammation. Additionally, AKG inhibits NF‐κB signaling, leading to reduced inflammatory gene expression, and dampens immune cell activation, including responses from macrophages and other immune cells, collectively contributing to its anti‐inflammatory action. Given that brain inflammation is closely linked to age‐related cognitive decline, the ability of CaAKG to reduce inflammatory markers could be a key mechanism behind its neuroprotective effects, particularly in females, ultimately contributing to its role in rescuing synaptic plasticity.

We could show that CaAKG ameliorates synaptic deficits in APP/PS1 mice in an NMDAR‐independent pathway. Application of AP‐5 did not affect the CaAKG‐mediated effects, which shows that CaAKG might modulate Ca^2+^ entry via NMDAR‐independent pathways. Accumulation of Aβ at early stages of the disease normally causes excessive activation of NMDARs that evokes an immediate Ca^2+^ rise, causing excitotoxicity (Liu et al. 2019). Therefore, blocking the effect on NMDARs or activating other Ca^2+^ sources for modulating intracellular Ca^2+^ levels could be a way to overcome synaptic deficits. Our results with CaAKG and nifedipine show that the voltage‐gated Ca^2+^ channels provide a major mechanism for activity‐induced Ca^2+^ entry into neurons, and blocking them prevents the rescue of LTP by CaAKG in AD mice. Although in WT slices, blocking LTCCs had no effect, showing that CaAKG induces NMDAR‐independent, LTCC‐dependent LTP in AD mice.

We also showed that CaAKG mediated effects occur via CP‐AMPARs in AD mice. Inhibition of CP‐AMPARs with IEM prevented the rescue of LTP by CaAKG in APP/PS1 mice, but did not affect LTP in WT mice. Ca^2+^ influx through CP‐AMPARs has been detected in multiple disease models (Guo and Ma 2021; Weiss 2011). CaAKG may promote activation of CP‐AMPARs in APP/PS1 mice, and this activity‐dependent insertion of CP‐AMPARs might lead to consolidation of LTP. This is similar to oleuropein‐enhanced LTP induction in 5XFAD mice, where GluA1 phosphorylation occurred in an NMDAR‐independent and CP‐AMPAR‐dependent manner (Wang et al. 2020). This suggests that enhanced CP‐AMPAR trafficking by CaAKG could be a novel target for facilitating synaptic plasticity and memory in AD. This is further supported by findings from pinoresinol, which induced NMDAR‐independent LTP that was blocked by IEM, a CP‐AMPAR antagonist, indicating that pinoresinol activated CP‐AMPAR‐dependent LTP in Aβ induced hippocampal slices (Yu et al. 2021).

Increased mTORC1 activity is another factor that accelerates the production of Aβ and accumulation of tau (Rapaka et al. 2022). Our studies show that inhibiting mTOR has a protective effect on synaptic plasticity in AD mice. The downregulation of mTOR signaling by rapamycin might trigger increased autophagy, and this could ameliorate synaptic deficits in APP/PS1 mice. More directly, rapamycin is known to reduce Aβ deposition, promote Aβ clearance, and inhibit hyper‐phosphorylation of tau protein (Hou et al. 2023). Thus, rapamycin might be especially useful before the onset of AD symptoms and during the early stages of the disease, making it a potential translational drug for early AD. This was confirmed by results from western blot showing that CaAKG treatment in AD mice increases the expression of autophagy markers.

We also showed that mTOR inhibition in WT mice suppressed late‐LTP unlike in APP/PS1 mice. This dichotomy is consistent with findings in aging models which demonstrated that mTOR inhibition blocks late‐LTP in young WT mice, whereas rescuing synaptic deficits in aged mice (Wang et al. 2023). In AD mice, the disrupted mRNA translation machinery due to excess mTOR activity might be regulated by rapamycin treatment, which reduces mTOR activity and enhances autophagy, resulting in the rescue of LTP. Inhibiting mTOR could lead to downregulation of protein synthesis and upregulation of autophagy in WT mice. In WT mice, rapamycin may suppress protein synthesis or enhance autophagy, disrupting cellular homeostasis and thereby blocking late‐LTP. These results also suggest that LTP in AD mice might be less sensitive to the effects of protein synthesis inhibition by rapamycin compared to the WT. The effects of CaAKG and rapamycin in enhancing autophagy may be additive or synergistic. We propose a model in which maintaining the proper balance between protein synthesis and degradation is essential at hippocampal synapses in WT mice. In APP/PS1 mice, however, mTOR overactivation suppresses autophagy, leading to reduced degradation of certain synaptic proteins. This highlights the role of autophagy in the aberrant synaptic plasticity and cognitive impairments observed in AD mice. These findings also suggest that other drugs which activate autophagy could also be promising therapeutics to improve synaptic function and cognitive outcomes in AD.

APP/PS1 mice show not only impaired LTP but also exhibit impaired associative plasticity/synaptic tagging and capture (STC) (Baby et al. 2020; Bin Ibrahim et al. 2024; Han et al. 2021; Krishna et al. 2020; Manakkadan et al. 2024; Pavon et al. 2023; Singh et al. 2022). We show here that CaAKG application reinstates STC in AD mice. CaAKG could facilitate the synthesis of new plasticity‐related proteins that can consolidate the late phase of LTP.

AKG has advantages over many other available AD drugs, as it is a naturally occurring compound in the body. Additionally, it has high solubility and stability in aqueous solutions, is fully absorbed by the body, and can cross the blood brain barrier. A supplementation protocol for AKG in middle‐aged adults has been developed and validated, demonstrating its feasibility and safety in humans. This supports the translational potential of AKG‐based interventions and provides a framework for future clinical studies (Sandalova et al. 2023).

## 5 Limitations

Our current ex vivo study demonstrated that CaAKG directly enhances synaptic plasticity in hippocampal slices; however, it did not assess behavioral outcomes or systemic pharmacokinetics. We also acknowledge that the effects of exogenously administered CaAKG may differ from those of endogenous AKG. Future studies will address these limitations by administering CaAKG orally in Alzheimer's disease mouse models, coupled with behavioral analyses and brain CaAKG quantification. These investigations are essential to determine the therapeutic relevance and mechanistic underpinnings of CaAKG under physiological conditions.

## Author Contributions

S.N. and B.K.K. conceived and coordinated the study. S.N. and B.K.K. wrote the paper. S.N. performed and analyzed the experimental data.

## Conflicts of Interest

The authors declare no conflicts of interest.

## Supporting information


**Figure S1:** Control experiments showing an impaired LTP in APP/PS1 mice with females having a faster decline than males. In hippocampal slices from male WT mice, the induction of late‐LTP by STET (A, *n* = 9) resulted in an immediate increase in fEPSP (1 min, *p* = 0.0010, Wilcoxon test; *p* = 0.0001, *U* test) that remained statistically significant at 240 min (*p* = 0.0010, Wilcoxon test; *p* = 0.0001, *U* test). In WT females (B, *n* = 8) also STET resulted in an immediate increase in fEPSP (1 min, *p* = 0.0156, Wilcoxon test; *p =* 0.0006, *U* test) that remained statistically significant at 240 min (240 min, *p* = 0.0156, Wilcoxon test; *p =* 0.0023, *U* test). In APP/PS1 males, STET resulted in potentiation that was significant only until 165 min (*p =* 0.0312, Wilcoxon test; *p* = 0.0087, *U* test) after which it decayed to the baseline (C, *n* = 6). In APP/PS1 females, STET led to a potentiation that remained stable only until 50 min, after which it declined to baseline from 55 min onward (50 min, *p* = 0.0234, Wilcoxon test; 55 min, *p* = 0.0781, Wilcoxon test) when compared to its own baseline (D, *n* = 7). It was significant until 50 min and declined to baseline at 55 min (50 min, *p* = 0.0104, *U* test test; 50 min, *p* = 0.0830, *U* test), when compared to the control S2. These results confirm the previous findings that APP/PS1 mice displayed impaired LTP and that female APP/PS1 mice exhibited a faster decline in LTP compared to male APP/PS1 mice.

## Data Availability

The data that support the findings of this study are available from the corresponding author upon request.
