# Supplementary figures and images for: Alpha‐Ketoglutarate Ameliorates Synaptic Plasticity Deficits in APP/PS1 Mice Model of Alzheimer's Disease
Source: Aging Cell. 2025 Sep 17;24(11):e70235. doi: 10.1111/acel.70235 (PMC12610948; doi:10.1111/acel.70235)

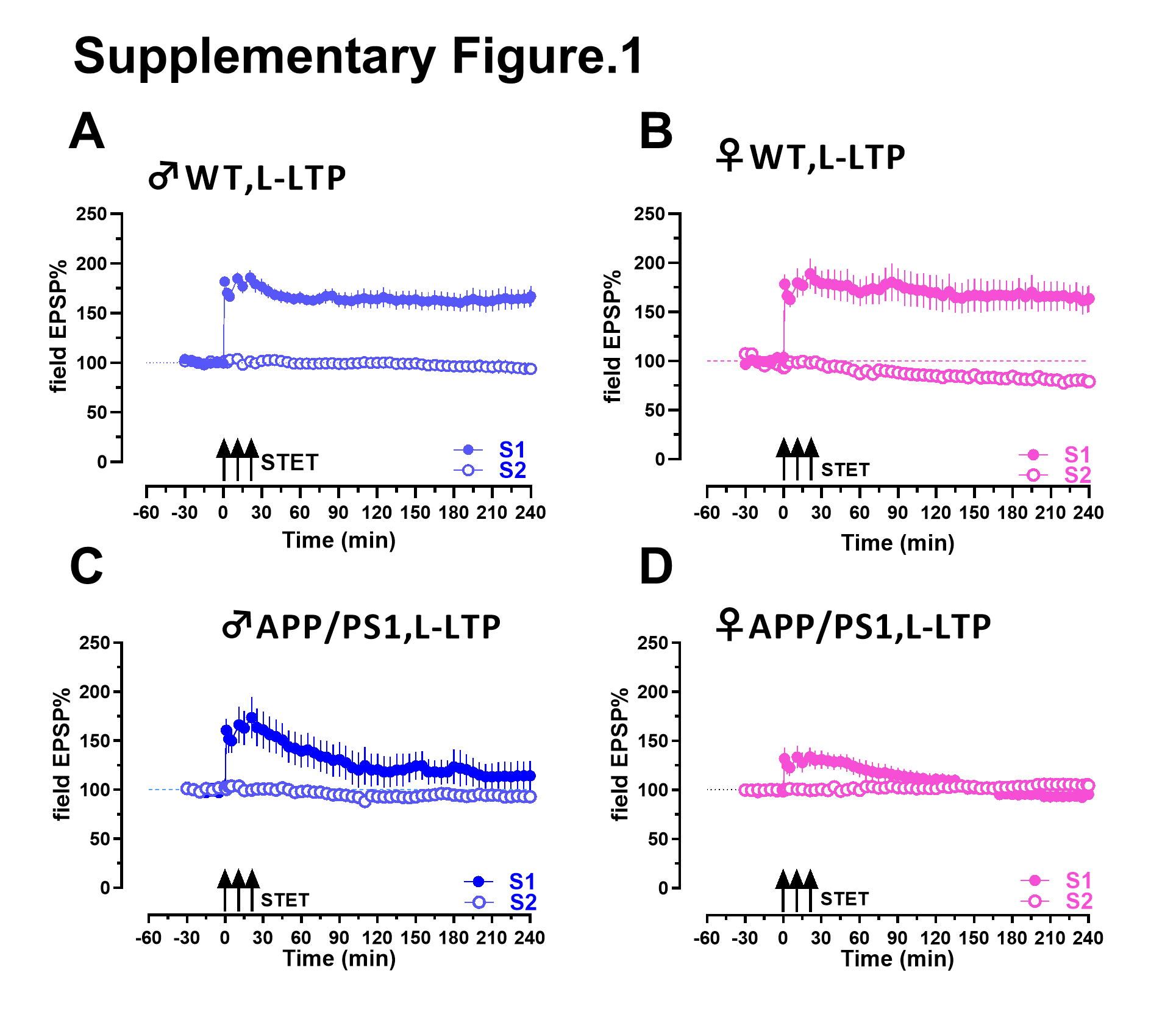

Supplement: Supplementary file 1 — Figure S1: Control experiments showing an impaired LTP in APP/PS1 mice with females having a faster decline than males. In hippocampal slices from male WT mice, the induction of late‐LTP by STET (A, n = 9) resulted in an immediate increase in fEPSP (1 min, p = 0.0010, Wilcoxon test; p = 0.0001, U test) that remained statistically significant at 240 min (p = 0.0010, Wilcoxon test; p = 0.0001, U test). In WT females (B, n = 8) also STET resulted in an immediate increase in fEPSP (1 min, p = 0.0156, Wilcoxon test; p = 0.0006, U test) that remained statistically significant at 240 min (240 min, p = 0.0156, Wilcoxon test; p = 0.0023, U test). In APP/PS1 males, STET resulted in potentiation that was significant only until 165 min (p = 0.0312, Wilcoxon test; p = 0.0087, U test) after which it decayed to the baseline (C, n = 6). In APP/PS1 females, STET led to a potentiation that remained stable only until 50 min, after which it declined to baseline from 55 min onward (50 min, p = 0.0234, Wilcoxon test; 55 min, p = 0.0781, Wilcoxon test) when compared to its own baseline (D, n = 7). It was significant until 50 min and declined to baseline at 55 min (50 min, p = 0.0104, U test test; 50 min, p = 0.0830, U test), when compared to the control S2. These results confirm the previous findings that APP/PS1 mice displayed impaired LTP and that female APP/PS1 mice exhibited a faster decline in LTP compared to male APP/PS1 mice. [file ACEL-24-e70235-s001.tif]
